# Supplementary material for: Multi-centric origins and gene flow shape the diversity of β-thalassemia mutations in Southern East Asia
Source: Nat Commun. 2025 Nov 20;16:10220. doi: 10.1038/s41467-025-65019-0 (PMC12635081; doi:10.1038/s41467-025-65019-0)
Supplement: Supplementary file 1 — Supplementary Information [file 41467_2025_65019_MOESM1_ESM.pdf]

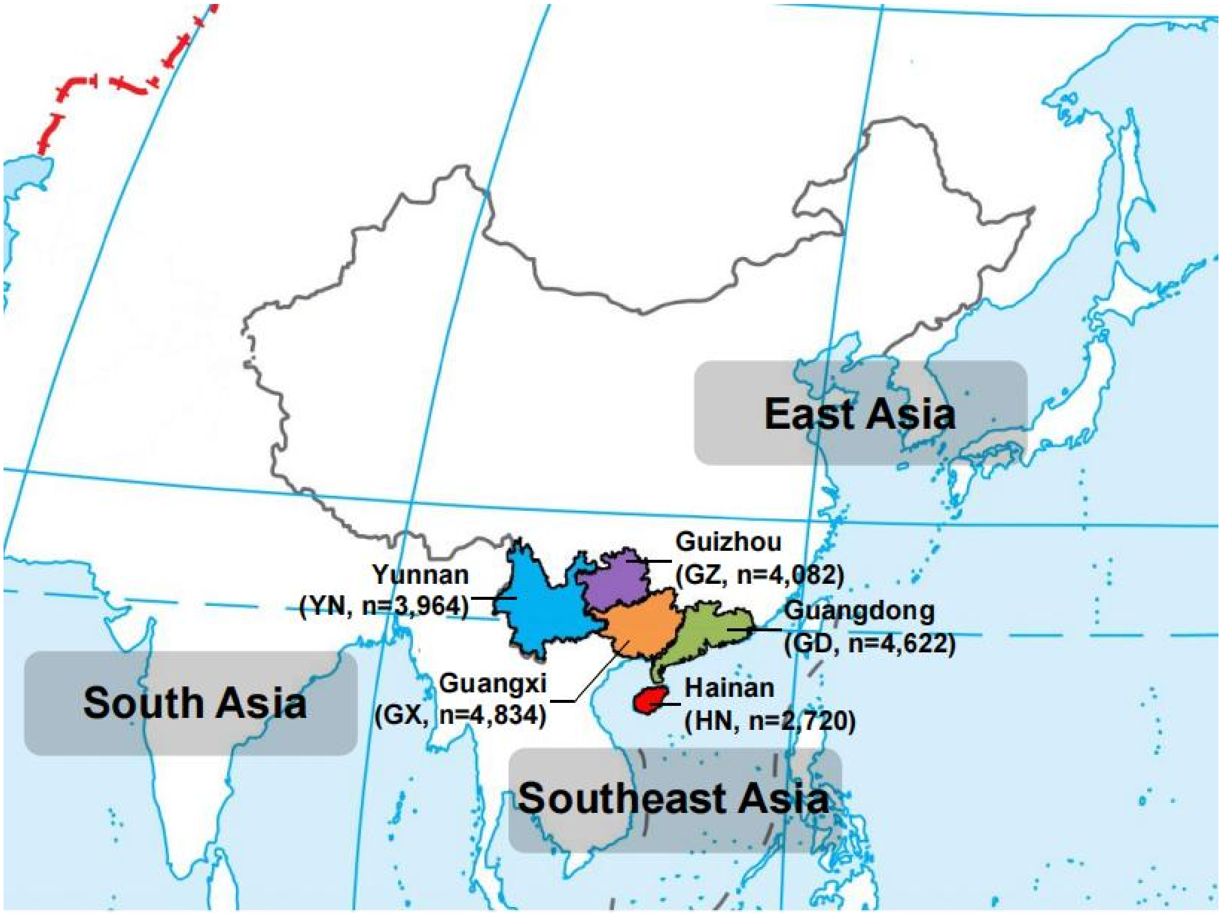

Supplementary Fig. 1 Map of the locations of the five provinces where samples were collected. Abbreviations of the provinces and sample sizes are shown in brackets. Situated at the crossroads of East, South, and Southeast Asia, these regions have historically served as corridors for human migration, cultural exchange, and pathogen spread. Their geographic and demographic diversity offers a valuable context for investigating the origin and evolutionary dynamics of β-thalassemia mutations in Chinese populations. The map is adapted from the issued version (No. GS(2016)2962) of the Ministry of Natural Resources of China, downloaded from the Standard Map Service Website (http://bzdt.ch.mnr.gov.cn).

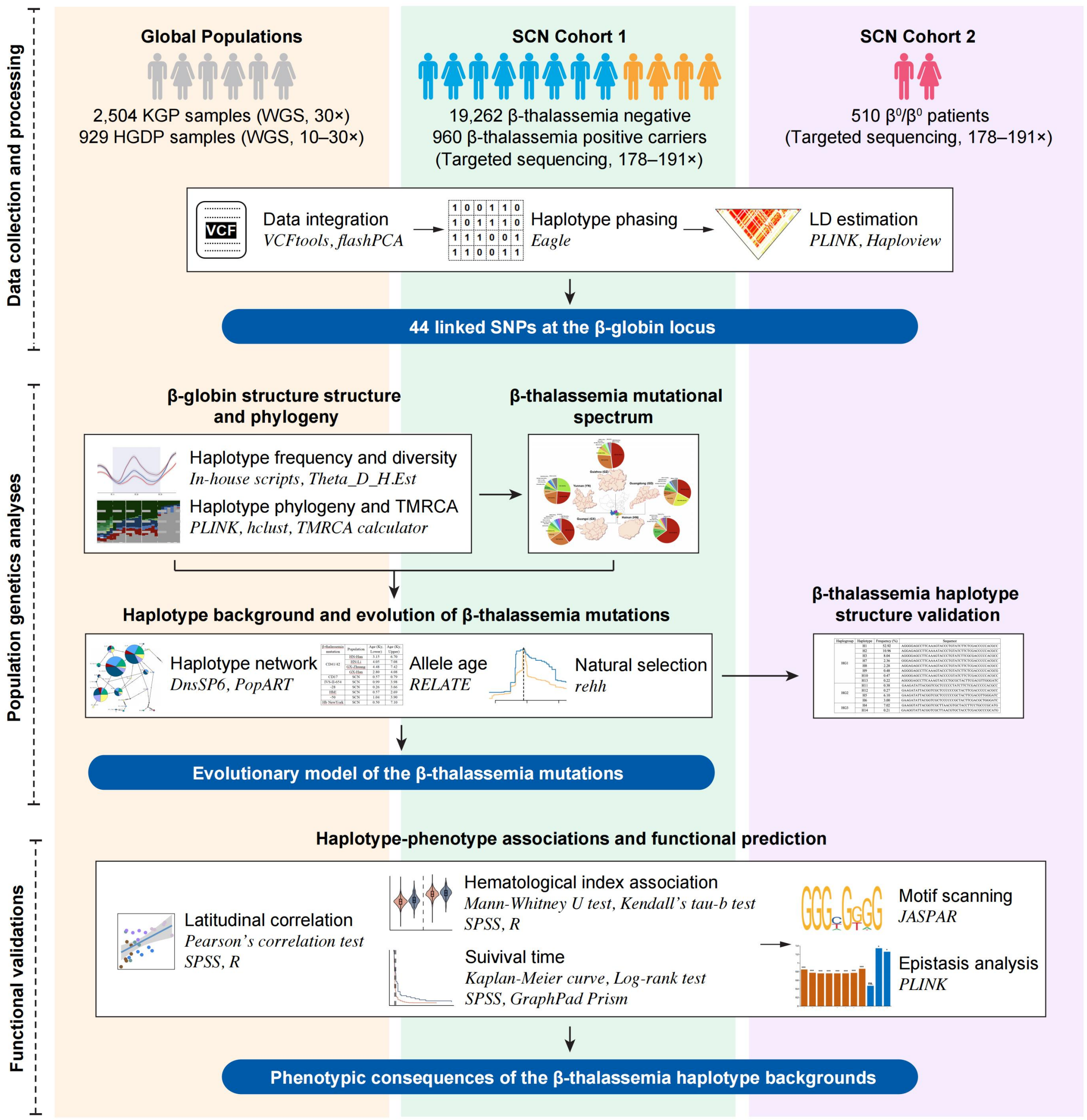

Supplementary Fig. 2 Integrative analytical framework of the study. This figure summarizes the multi-layered analytical approach employed, integrating genome-wide and targeted sequencing data from global populations (KGP and HGDP) and two southern Chinese cohorts (SCN Cohort 1: n = 19,282; SCN Cohort 2: n = 510 patients), respectively. A total of 44 linked SNPs in the β-globin locus were analyzed to investigate the haplotype structure and evolutionary dynamics of the β-thalassemia mutations, with phylogenetic analyses, allele age estimation, and adaptation inference. Functional assessments were performed to evaluate the phenotypic impact of different haplotype backgrounds, including hematological indices, survival analyses, and motif/epistasis predictions. Collectively, this integrative pipeline establishes an evolutionary model of β-thalassemia and elucidates its phenotypic consequences across diverse populations.

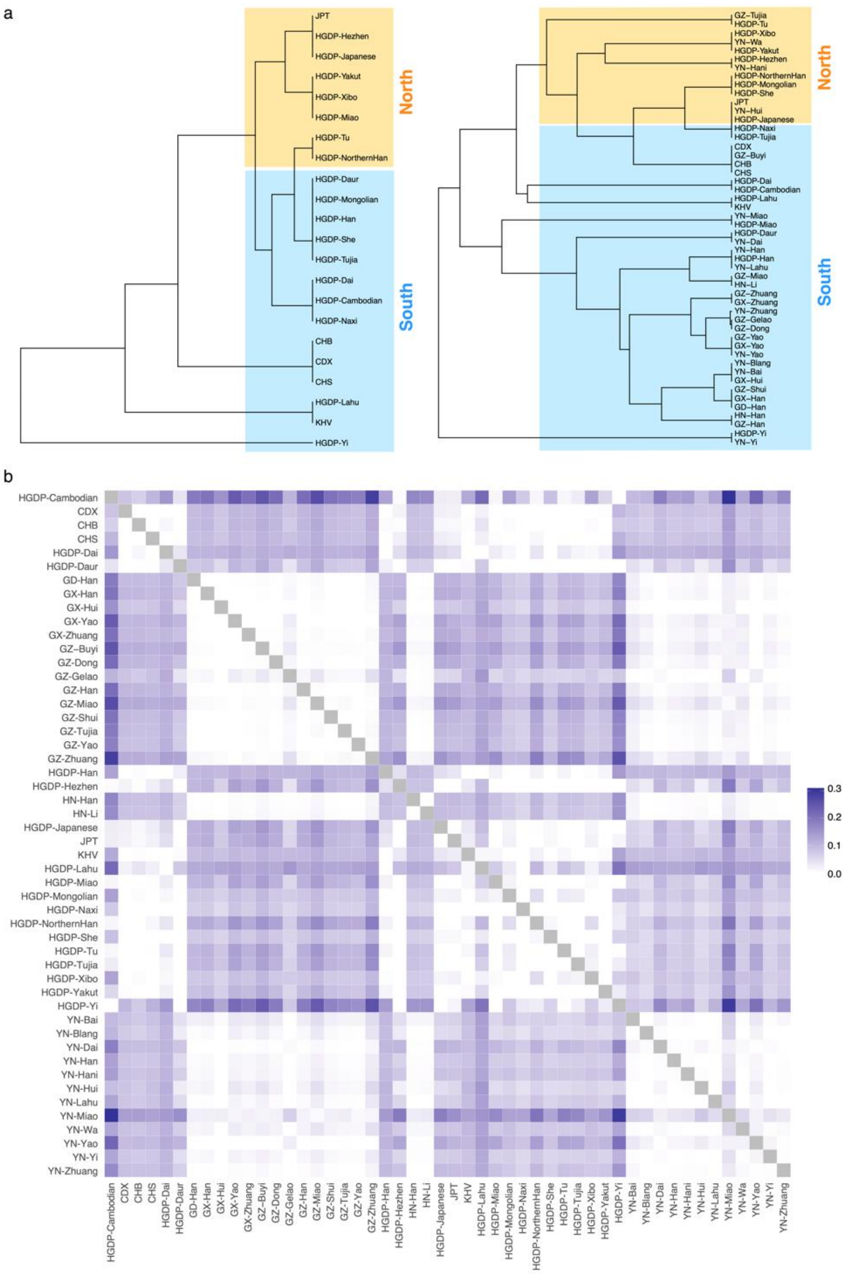

Supplementary Fig. 3 Genetic differentiation and population structure among SCN and global populations. a UPGMA trees constructed using pairwise F_{ST} values illustrate population genetic relationships at the β-globin locus, based on the population data from the 1000 Genomes Project (KGP), Human Genome Diversity Panel (HGDP), and Southern Chinese (SCN). Northern populations (orange) and southern populations (blue) form distinct clusters. Consistent ethnic groups across datasets showed a tendency to cluster. **b** Heatmap of F_{ST} values showing the degree of genetic differentiation in the β-globin locus among populations from KGP, HGDP, and SCN. Color intensity corresponds to genetic differentiation levels. SCN populations show clear regional substructure and exhibit greater genetic affinity to Southeast Asian groups (e.g., Dai, Cambodian) than to northern East Asians, supporting a geographically structured distribution of β-thalassemia in southern China.

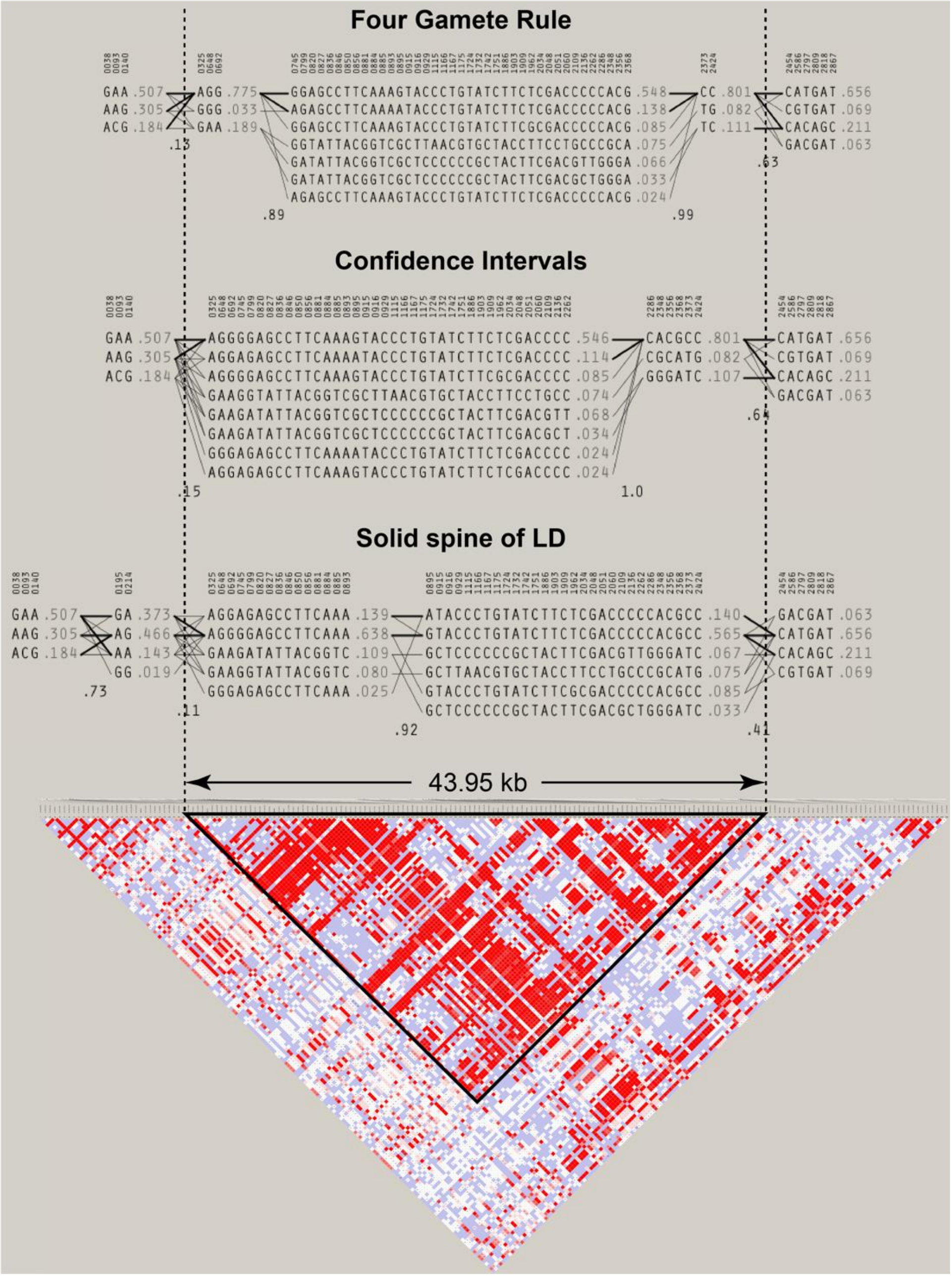

Supplementary Fig. 4 Linkage disequilibrium (LD) at the β-globin locus in 20,222 southern Chinese samples. LD was evaluated using three algorithms: Four Gamete Rule, Confidence Intervals, and Solid Spine of LD. All methods consistently identified a strong LD block spanning ~43.95 kb, indicating a robust and conserved haplotype structure at the β-globin locus. Forty-four SNPs with a minor allele frequency > 5% are shown in the haplotype sequence. The concordance across algorithms supports the reliability of this LD block, which served as the foundation for haplotype reconstruction and evolutionary inference in subsequent analyses.

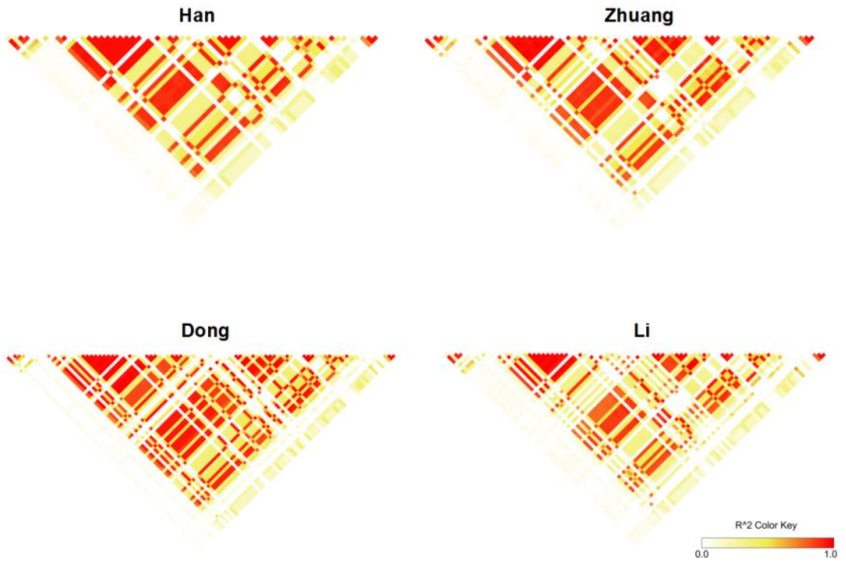

Supplementary Fig. 5 LD structure at the β-globin locus across five major ethnic groups in southern China. LD blocks are shown for Han Chinese (n = 13,485), Zhuang (n = 1,936), Miao (n = 733), Dong (n = 627), and Li (n = 558) populations within the SCN cohort. Despite differences in linguistic affiliation and geographic origin, all groups display broadly similar LD patterns, characterized by conserved regions of high pairwise r^2 values across the locus. The shared LD block across ethnolinguistically diverse groups in southern China supports the use of a unified LD block in subsequent evolutionary and association analyses.

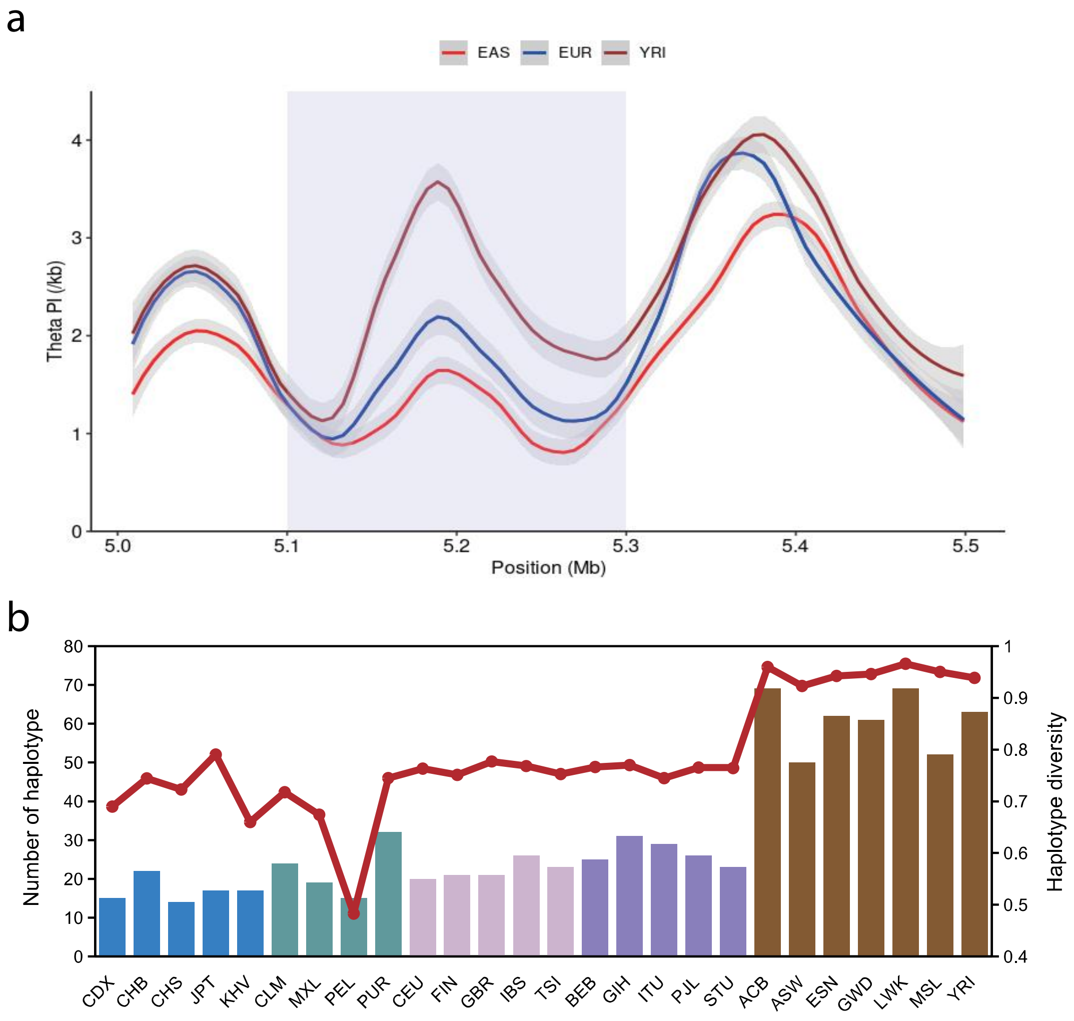

Supplementary Fig. 6 Genetic diversity at the β-globin LD block across global populations. a Nucleotide diversity at the β-globin locus. θ_π was estimated in sliding windows of 50 kb, advanced by 10 kb, in three continental groups from the 1000 Genomes Project (EAS: red; EUR: blue; YRI: black). The shaded area highlights the LD block encompassing the β-globin locus region, where East Asians exhibit markedly reduced nucleotide diversity relative to Europeans and Africans, suggesting population-specific bottlenecks or historical selective sweeps. **b** Haplotype diversity across global populations from the KGP dataset. The number of unique haplotypes (bars, left y-axis) and haplotype diversity (connected dots, right y-axis) are shown for each population. Source data are provided as a Source Data file.

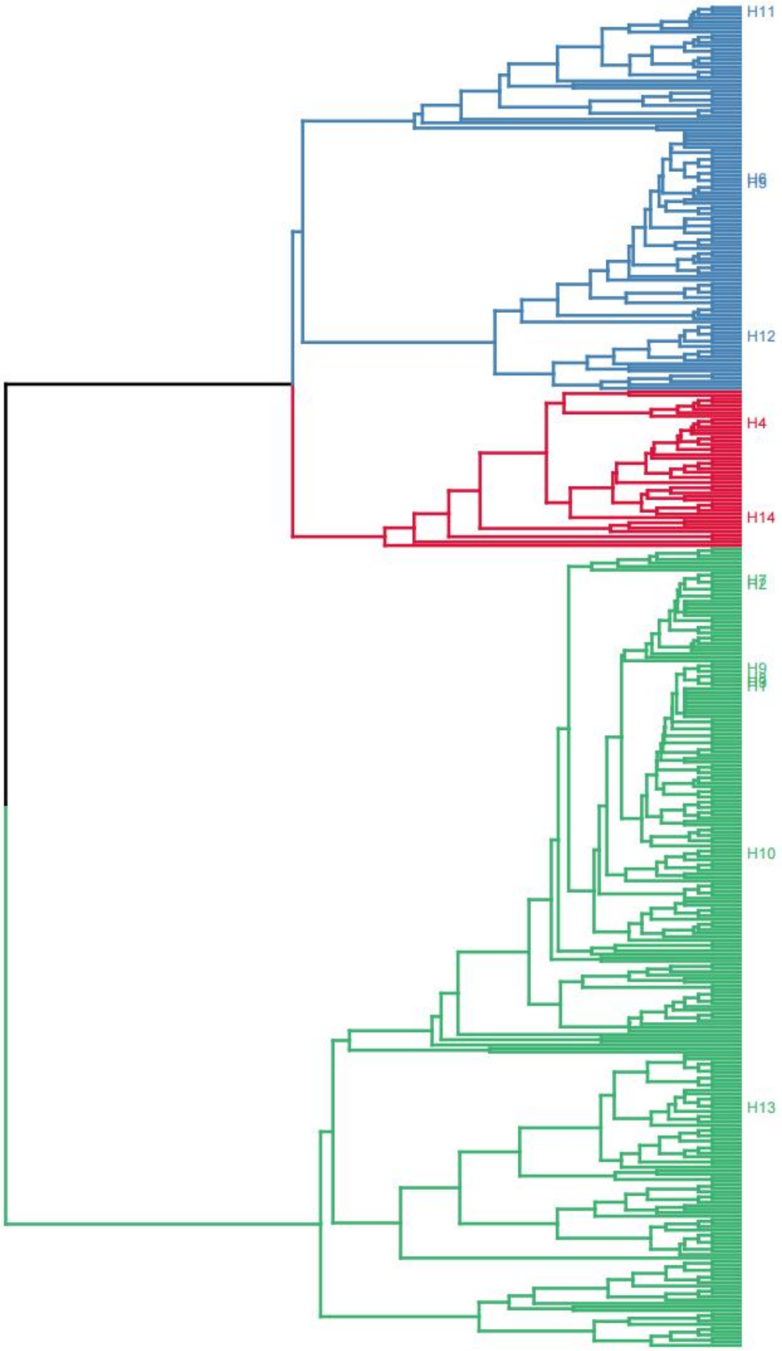

Supplementary Fig. 7 Hierarchical clustering of β-globin haplotypes in the SCN samples. A total of 384 haplotypes were reconstructed from 44 common SNPs (minor allele frequency > 5%) spanning the β-globin LD block in 20,222 individuals from the SCN cohort. The dendrogram depicts hierarchical clustering based on identity-by-state (IBS) distances, revealing three evolutionarily distinct haplogroups: HG1 (green), HG2 (blue), and HG3 (red). Fourteen high-frequency haplotypes (H1–H14; each > 0.2% frequency) are annotated and collectively account for 94.7% of all observed haplotypes.

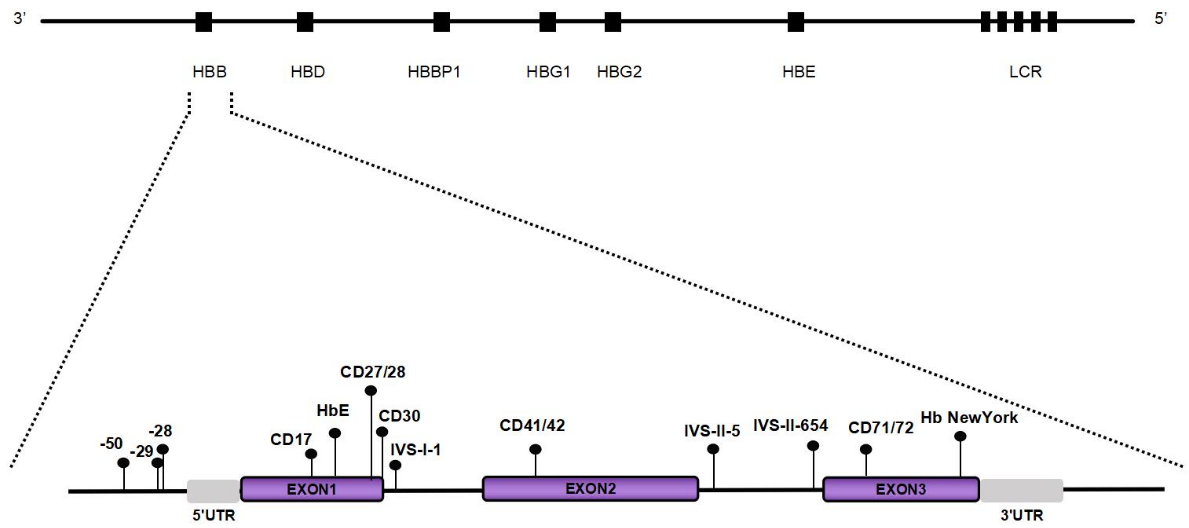

Supplementary Fig. 8 Genomic distribution of the 13 most prevalent β-thalassemia mutations in southern China. This schematic illustrates the β-globin locus, including *HBD, HBBP1, HBB, HBG1, HBG2, HBE*, and the locus control region (LCR), providing genomic context for common β-thalassemia mutations. A magnified inset of the *HBB* gene depicts its exon–intron structure and the precise locations of 13 recurrent pathogenic variants, which collectively account for ~95% of β-thalassemia cases in southern China. These mutations are distributed across multiple functional elements, including promoter regions (e.g., −50, −28), untranslated regions (UTRs), coding exons (e.g., CD17, CD27/28, CD41/42), and canonical splice sites (e.g., IVS-I-1, IVS-II-654). Their genomic distribution reflects both functional constraints and historical founder effects, underscoring their clinical relevance for molecular screening and diagnosis in this population. Genomic coordinates and allele frequencies are detailed in Supplementary Data 1.

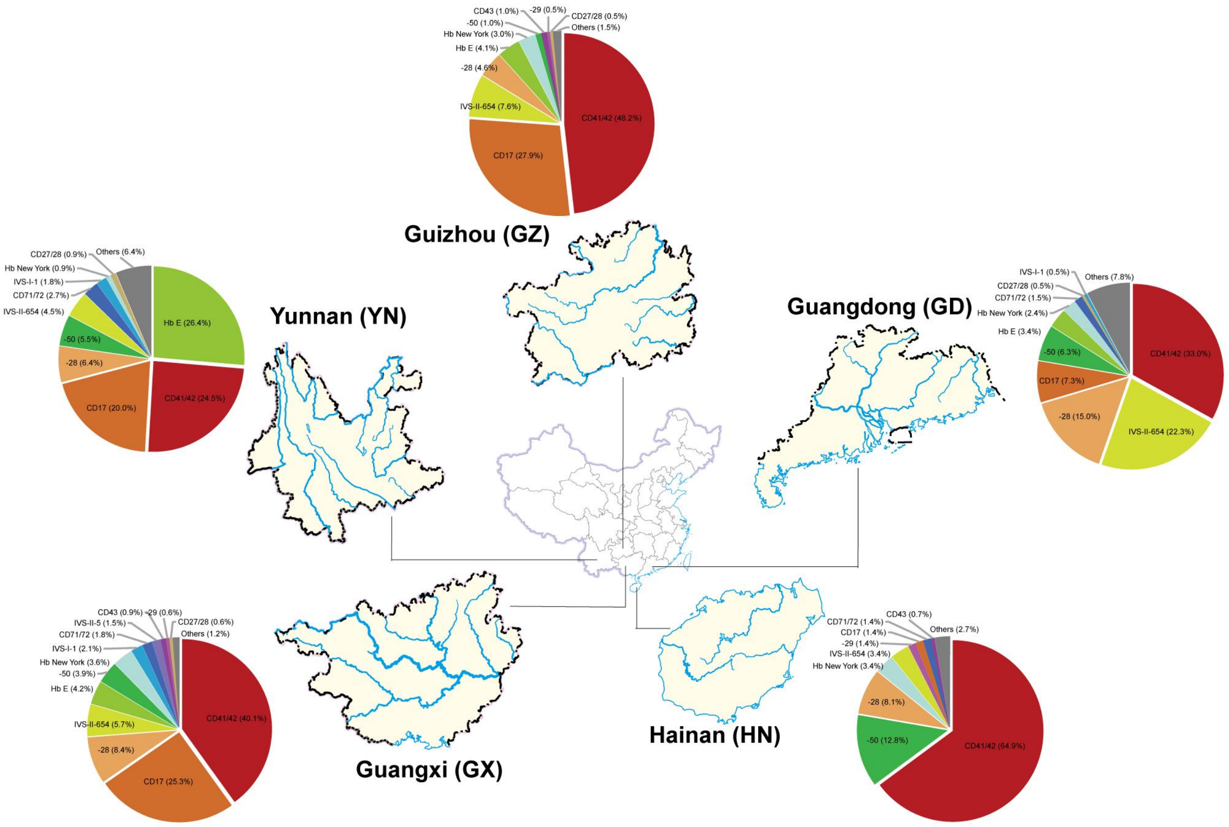

Supplementary Fig. 9 Geographic distribution of the 13 most prevalent β-thalassemia mutations across southern China. Pie charts illustrate the regional composition of β-thalassemia mutations based on epidemiological screening data from five provinces: Guangdong (GD), Guangxi (GX), Yunnan (YN), Guizhou (GZ), and Hainan (HN). Each segment represents the relative frequency of one of the 13 most common pathogenic variants. While core mutations such as CD41/42 and CD17 are shared across all regions, their frequencies vary substantially, reflecting region-specific genetic architectures. Notably, the Hainan population displays a pronounced enrichment of CD41/42 (64.9% of cases), suggesting reduced mutational diversity likely shaped by founder effects and demographic isolation. These findings underscore the importance of regionally tailored genetic screening and intervention strategies. Full mutation names and allele frequencies are provided in Supplementary Data 1. Source data are provided as a Source Data file. The map is adapted from the issued version (No. GS(2019)1673 and GS(2019)3333) of the Ministry of Natural Resources of China, downloaded from the Standard Map Service Website (http://bzdt.ch.mnr.gov.cn).

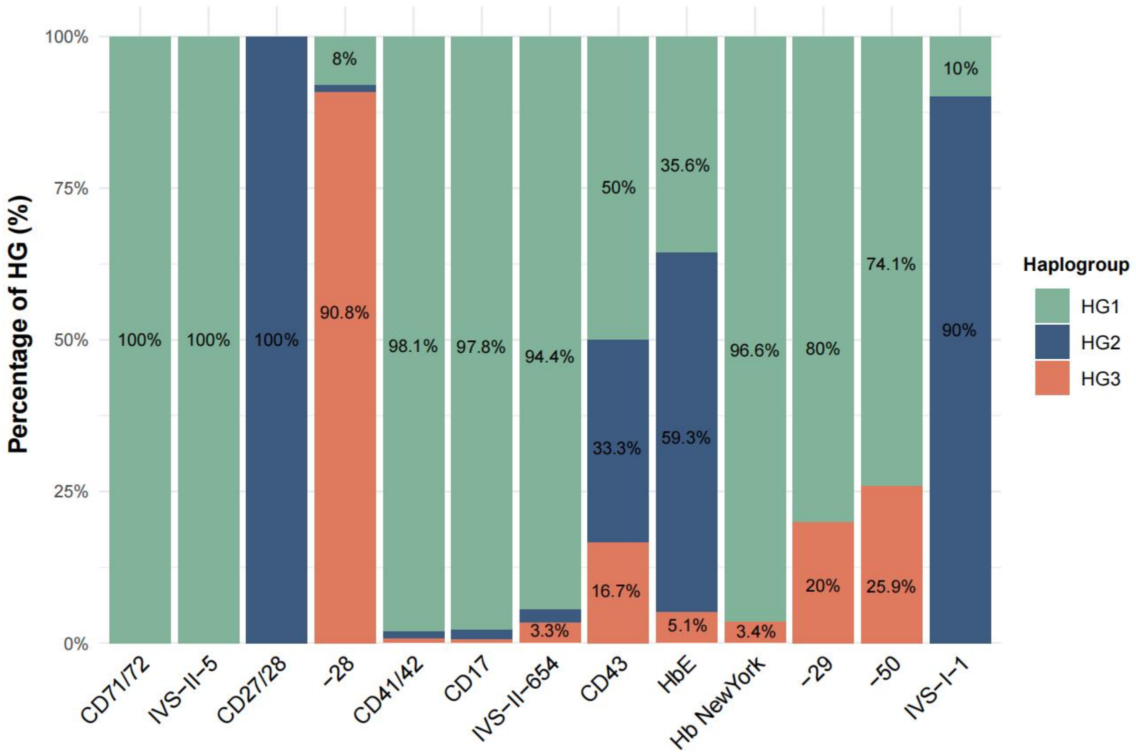

Supplementary Fig. 10 Haplotype composition of β-thalassemia mutations in southern China. Bar plots show the distribution of three major haplogroups (HG1–HG3) linked to each of the 13 most prevalent β-thalassemia mutations among 960 carriers in the Southern Chinese (SCN) cohort. Mutations such as CD71/72, IVS-II-5, and CD27/28 exhibit near-exclusive associations with a single haplogroup (primarily HG1), indicative of limited haplotypic diversity and possible recent origin. In contrast, mutations including CD41/42, CD17, and HbE span all three haplogroups, suggesting multiple ancestral backgrounds or recurrent gene flow. Notably, the −28 mutation is almost exclusively linked to HG3, especially haplotype H14, highlighting a potential founder effect or localized selective sweep. These findings reveal mutation-specific haplotype architectures at the β-globin locus and provide a framework for understanding the evolutionary and clinical dynamics of β-thalassemia alleles in diverse southern Chinese populations. Source data are provided as a Source Data file.

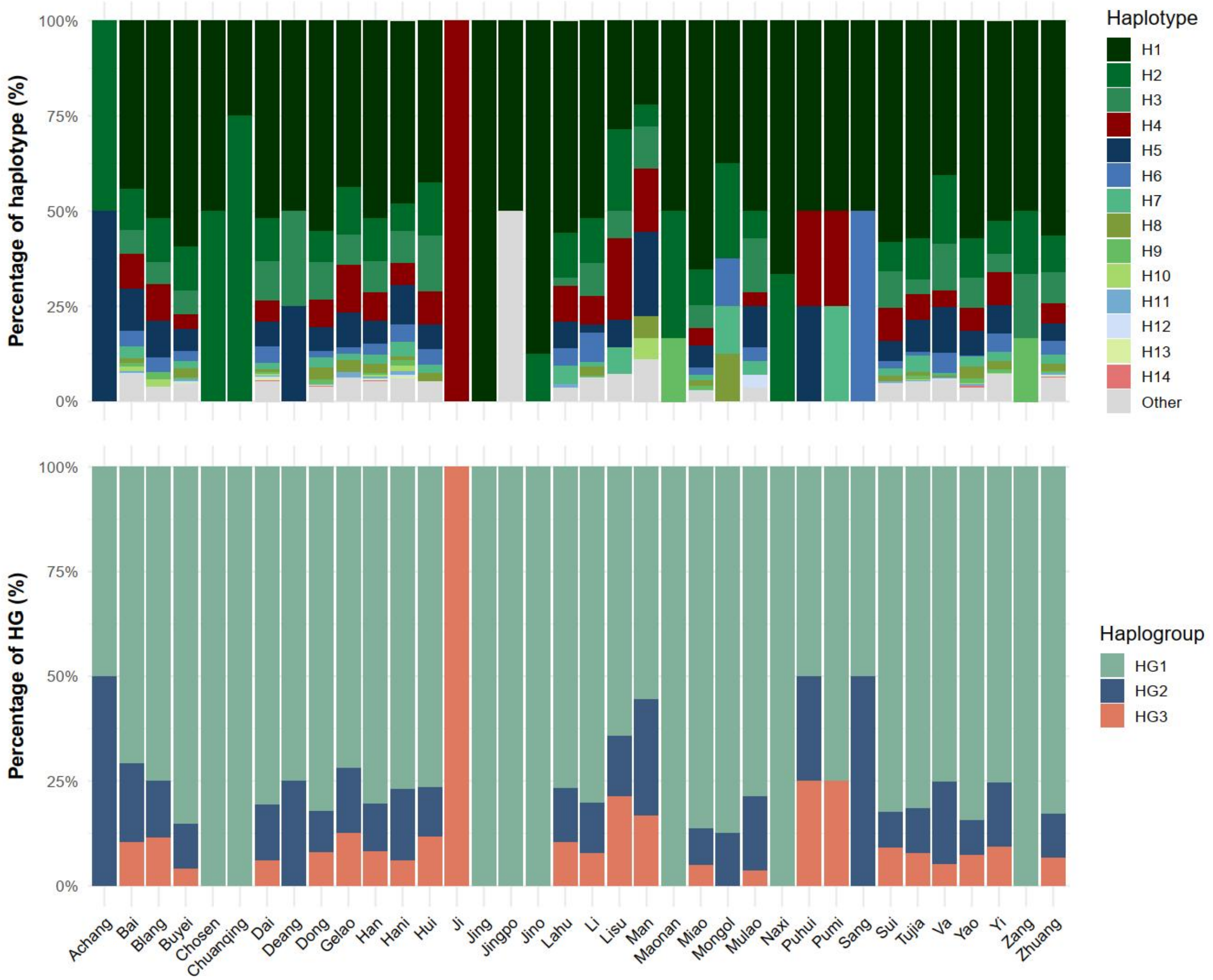

Supplementary Fig. 11 Haplotype composition of the β-thalassemia mutations across ethnic populations in southern China. Each bar represents the composition of haplotypes (upper) and haplogroups (lower) within different ethnic groups. While a broadly conserved haplotype structure is observed across populations from different provinces, notable inter-ethnic variation exists in the frequency and composition of specific haplotypes and haplogroups. Source data are provided as a Source Data file.

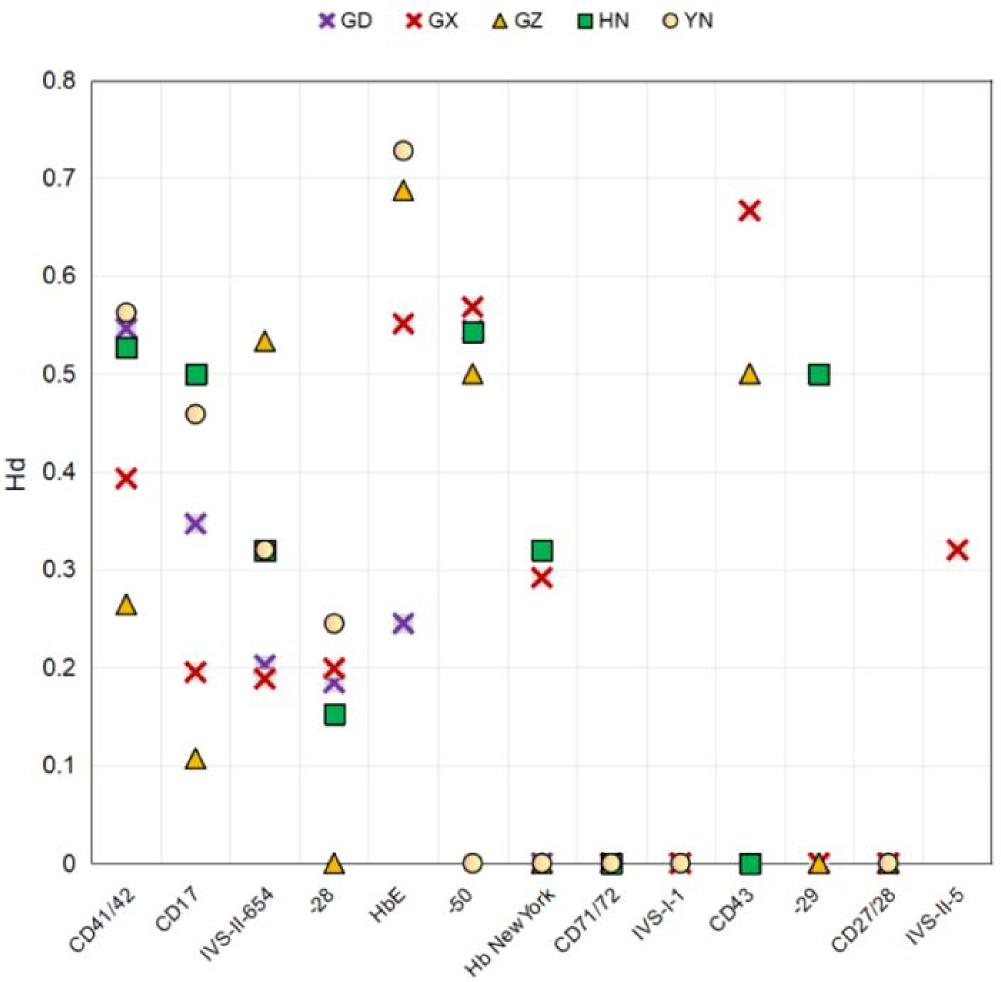

Supplementary Fig. 12 Haplotype diversity of 13 β-thalassemia mutations across five provinces in southern China. Each point represents the haplotype diversity (Hd) of a specific β-thalassemia mutation within a single province. Pronounced regional heterogeneity is observed, with mutations such as CD41/42, HbE, and –50 displaying substantially higher haplotype diversity compared to others. Notably, HbE exhibits exceptional diversity in Yunnan, likely reflecting the province's pronounced ethnic and genetic complexity. Conversely, low-frequency variants such as CD27/28 and IVS-II-5 show minimal haplotype diversity, suggesting potential founder effects or recent emergence. These patterns highlight distinct regional evolutionary trajectories of β-globin mutations and underscore the relevance of local genetic backgrounds for precision screening and disease-mapping strategies. Source data are provided as a Source Data file.

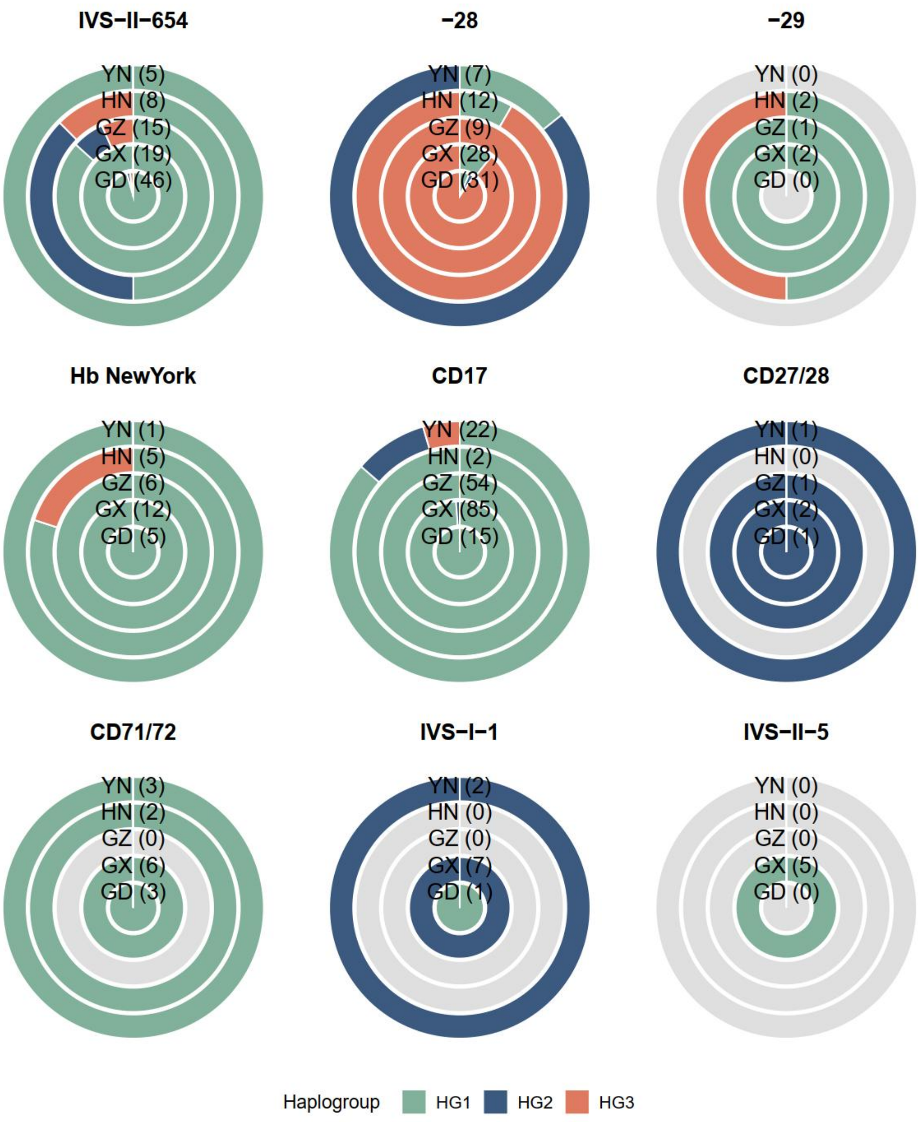

Supplementary Fig. 13 Haplotype composition of β-thalassemia mutations across five southern Chinese provinces. Donut plots illustrate the distribution of three major haplogroups (HG1: green, HG2: blue, HG3: red) associated with nine β-thalassemia mutations in Guangdong (GD), Guangxi (GX), Yunnan (YN), Guizhou (GZ), and Hainan (HN). Numbers in parentheses denote the number of mutation carriers per province. While some mutations, such as CD17, CD71/72, and CD27/28, are predominantly confined to a single haplogroup (primarily HG1 or HG2), others like –28 and Hb NewYork show broader haplogroup distributions, particularly in genetically diverse provinces such as Yunnan and Hainan. IVS-II-5 is detected in only one individual from Guangdong, highlighting its rarity. CD17, although widespread geographically, remains consistently associated with HG1. Source data are provided as a Source Data file.

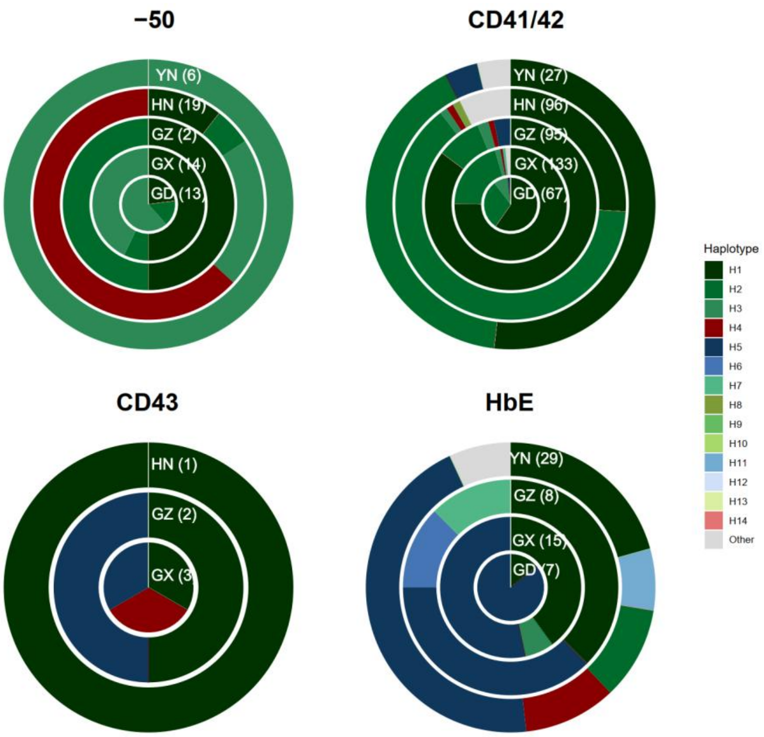

Supplementary Fig. 14 Haplotype distribution of four β-thalassemia mutations with the highest haplotype diversity across five provinces in southern China. Donut plots illustrate the haplotype composition of mutations –50, CD41/42, CD43, and HbE in five provinces. Colors represent haplotypes (H1–H14 and others), with sample sizes indicated in parentheses. Each mutation displays distinct haplotype patterns across regions.

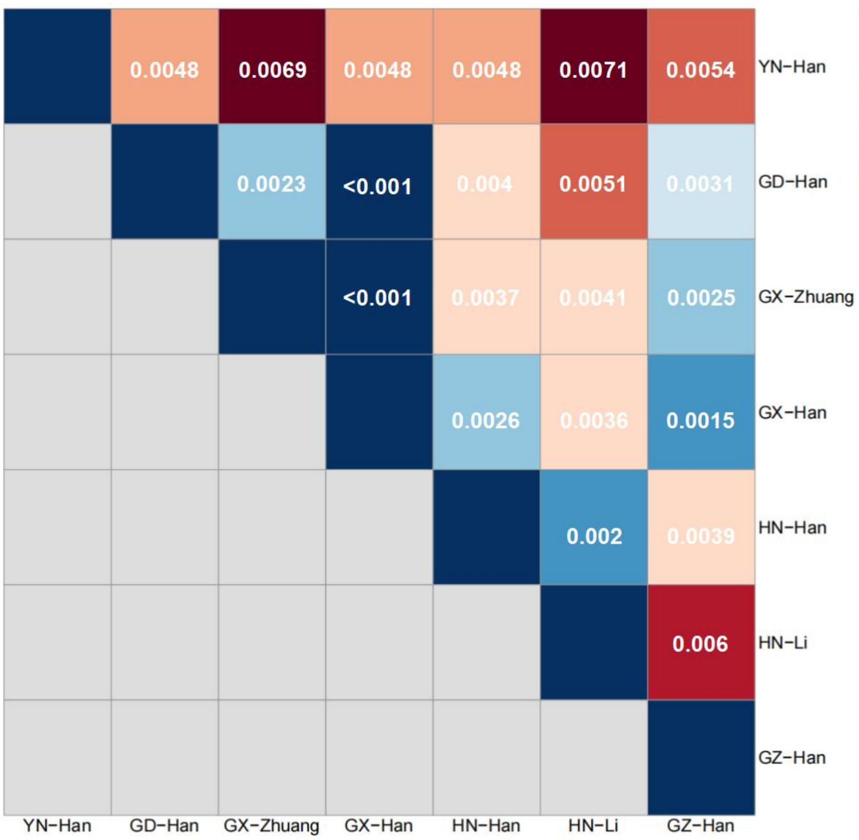

Supplementary Fig. 15 Pairwise genetic differentiation F_{ST} value at the β-globin locus among major southern Chinese populations. Heatmap shows F_{ST} values based on haplotypes at the β-globin locus, calculated between Han Chinese from Yunnan (YN), Guangdong (GD), Guangxi (GX), Hainan (HN), and Guizhou (GZ), the Zhuang population from Guangxi (GX-Zhuang), and the Li population from Hainan (HN-Li).

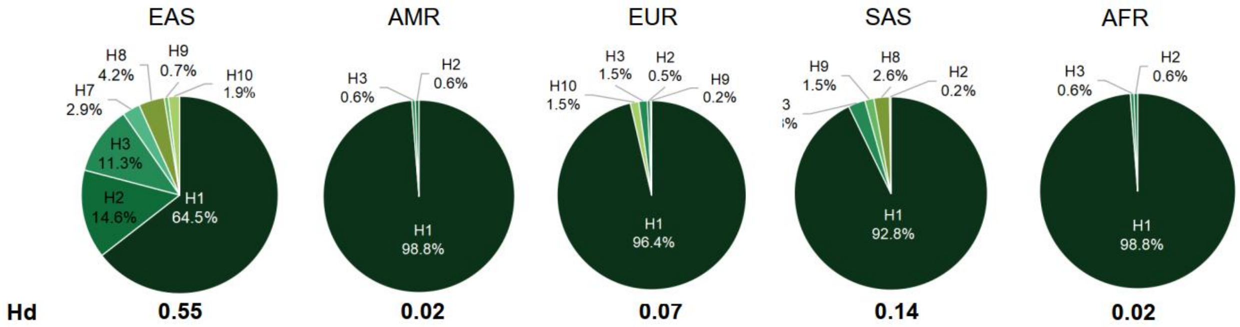

Supplementary Fig. 16 Subhaplotype composition and diversity within HG1 across continental populations. Pie charts depict the relative proportions of subhaplotypes within haplogroup 1 (HG1) in five continental populations from the 1000 Genomes Project: East Asians (EAS), Americans (AMR), Europeans (EUR), South Asians (SAS), and Africans (AFR). Haplotype diversity (Hd) within HG1 is shown below each chart. East Asian populations exhibit markedly higher subhaplotype diversity within HG1 compared to other continental groups, suggesting region-specific haplotype expansion.

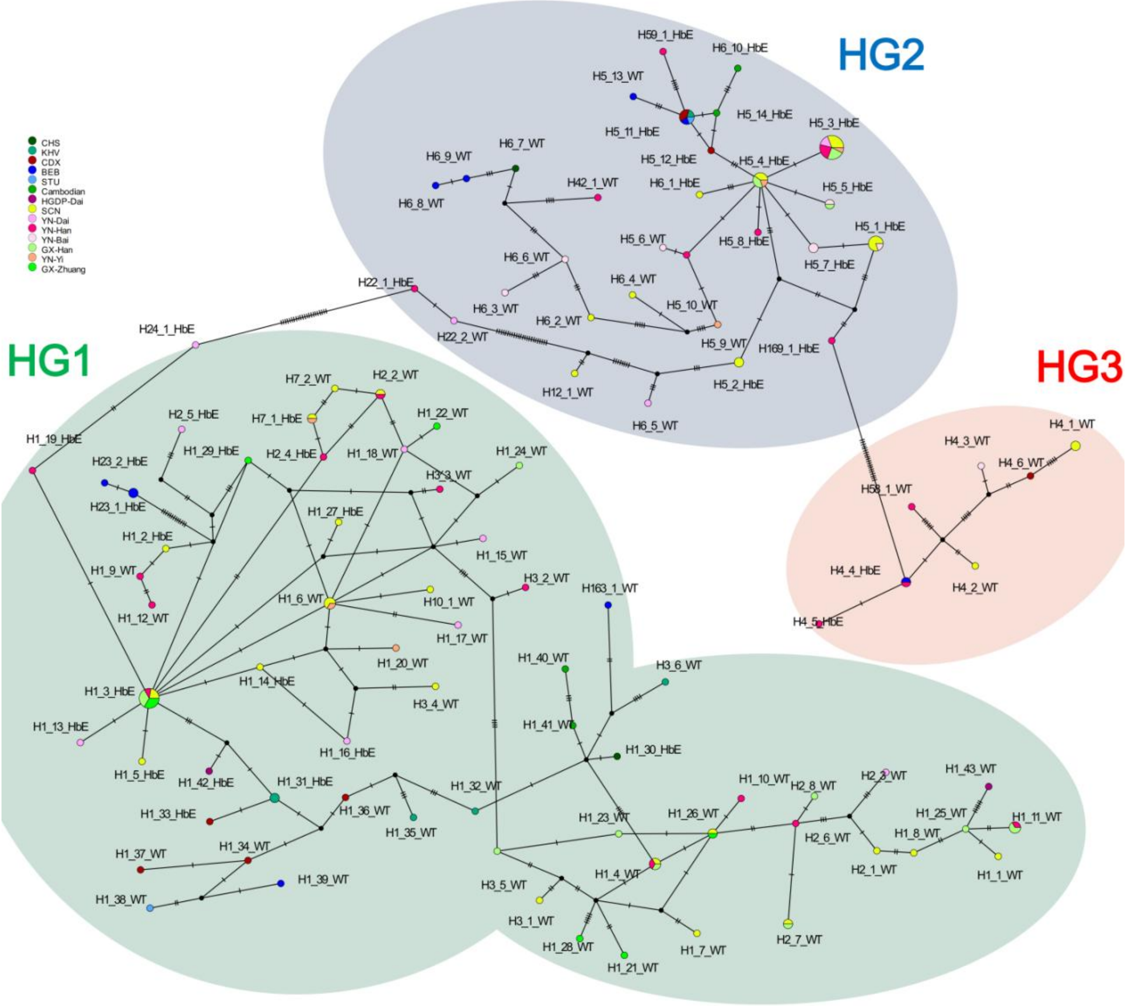

Supplementary Fig. 17 Expanded haplotype network of HbE carriers across South China and global populations. Median-joining haplotype network constructed using extended SNPs spanning the β-globin locus among individuals carrying the HbE mutation from the Southern Chinese Network (SCN), the 1000 Genomes Project, and the Human Genome Diversity Project (HGDP). Each node represents a unique haplotype, with node size proportional to the number of individuals sharing that haplotype; colors indicate population origin. Edges correspond to single nucleotide differences between haplotypes. Haplotypes are grouped into three major haplogroups, HG1 (green), HG2 (blue), and HG3 (red). HbE-associated haplotypes from SCN populations are centrally positioned within HG1 and HG2, consistent with ancestral or hub-like roles. In contrast, South and Southeast Asian haplotypes are found at peripheral branches, suggesting subsequent diversification events. Each node label includes the haplotype ID, subhaplotype index, and the HbE mutation status (WT: wild-type), separated by underscores.

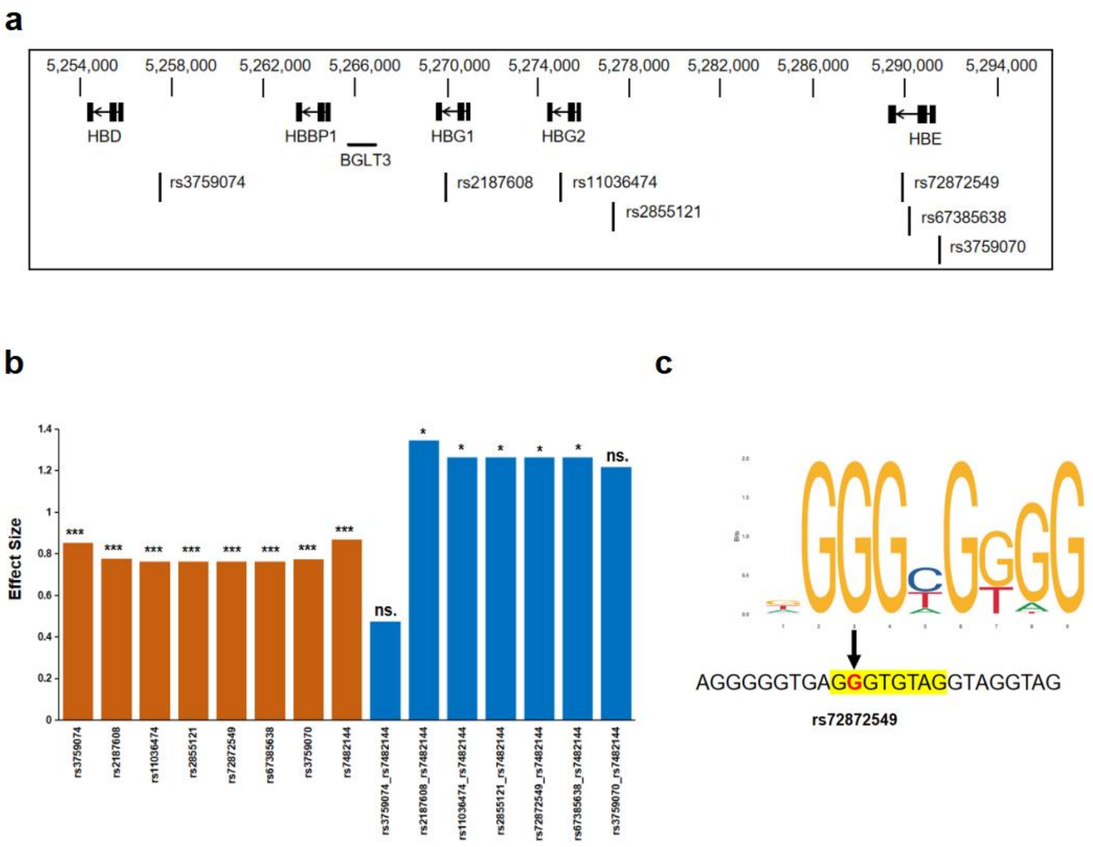

Supplementary Fig. 18 Association between HG2-specific mutations and HbF levels. **a** Genomic locations of seven HG2-specific mutations. **b** Effects of HG2-specific mutations and rs7482144 on HbF levels in β-thalassemia patients. The effect size of each SNP is represented by the brown bar, whereas the effect size of its interaction with rs7482144 is represented by the blue bar. Five of the seven variants show significant synergistic effects with rs7482144, a well-established regulatory SNP at the *HBG2* promoter, suggesting potential combinatorial regulation of γ-globin expression. **c** Predicted binding motif of KLF1, with rs72872549 located at the second base of the core motif. Source data are provided as a Source Data file.

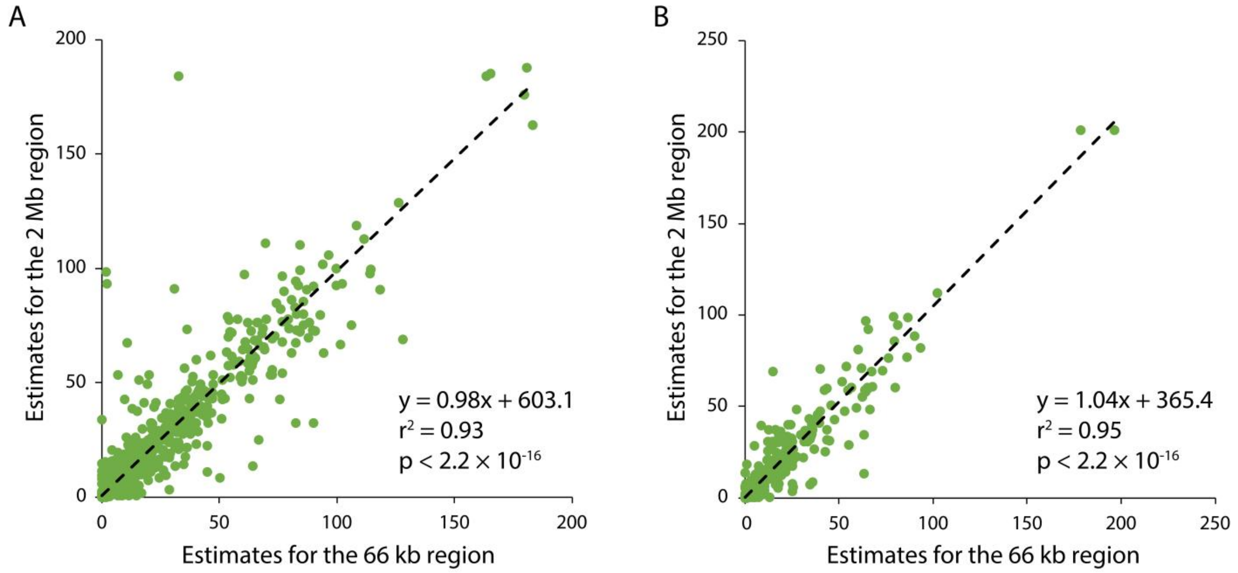

Supplementary Fig. 19 Positive correlation between allele age estimates based on different genomic region lengths. We assessed the impact of genomic region length on allele age estimates using East Asian genome data from the 1000 Genome Projects. Linear regression shows a significant correlation between the estimates derived from segments of varying lengths centered on the β-globin locus, with a median decrease of 0.08% in the estimates obtained from the 66 kb targeted region compared to those from the 2 Mb region. A two-sided Pearson correlation test (single comparison; no multiple-testing correction applied) was performed to obtain the p values.

Supplementary Table 1 Regional and ethnic distribution of 20,222 individuals from five provinces in southern China. The table summarizes the sample size of each ethnic population in each province, with counts provided for all samples (All) and for heterozygous carriers of β-thalassemia mutations without a β-thalassemia diagnosis (Carrier). The ethnic population IDs are from https://www.gov.cn/test/2006-04/04/content_244533.htm.

Ethnic Populations	Yunnan (YN)		Guizhou (GZ)		Guangxi (GX)		Guangdong (GD)		Hainan (HN)		Total
	All	Carrier	All	Carrier	All	Carrier	All	Carrier	All	Carrier	
Achang	1	0	0	0	0	0	0	0	0	0	1
Bai	275	6	0	0	0	0	0	0	0	0	275
Blang	26	2	0	0	0	0	0	0	0	0	26
Buyei	0	0	318	25	0	0	0	0	0	0	318
Chosen	0	0	1	0	0	0	0	0	0	0	1
Chuanqing	0	0	2	0	0	0	0	0	0	0	2
Dai	226	25	0	0	0	0	0	0	0	0	226
Deang	2	0	0	0	0	0	0	0	0	0	2
Dong	0	0	608	29	19	0	0	0	0	0	627
Gelao	0	0	32	0	0	0	0	0	0	0	32
Han	1,999	37	2,167	106	3,089	187	4,605	203	2,165	95	14,025
Hani	237	1	0	0	1	0	0	0	0	0	238
Hui	24	0	1	0	22	1	0	0	0	0	47
Ji	0	0	1	0	0	0	0	0	0	0	1
Jing	0	0	0	0	1	1	0	0	0	0	1
Jingpo	1	0	0	0	0	0	0	0	0	0	1
Jino	4	0	0	0	0	0	0	0	0	0	4
Lahu	42	1	0	0	0	0	1	0	0	0	43
Li	0	0	0	0	2	1	1	0	555	53	558
Lisu	7	0	0	0	0	0	0	0	0	0	7
Man	1	0	2	0	4	1	2	0	0	0	9
Maonan	0	0	0	0	3	0	0	0	0	0	3
Miao	215	2	499	17	17	0	2	0	0	0	733
Mongol	2	0	1	0	1	0	0	0	0	0	4
Mulao	0	0	0	0	14	1	0	0	0	0	14
Naxi	2	1	0	0	1	0	0	0	0	0	3
Puhui	2	0	0	0	0	0	0	0	0	0	2
Pumi	2	0	0	0	0	0	0	0	0	0	2
Sang	0	0	0	0	1	0	0	0	0	0	1
Sui	0	0	196	3	3	0	0	0	0	0	199
Tujia	4	0	148	5	10	0	0	0	0	0	162
Va	127	1	0	0	0	0	0	0	0	0	127
Yao	33	2	39	5	65	7	5	1	0	0	142
Yi	445	7	0	0	1	0	1	0	0	0	447
Zang	0	0	0	0	2	0	1	0	0	0	3
Zhuang	287	25	67	7	1,578	133	4	2	0	0	1,936
Total	3,964	110	4,082	197	4,834	332	4,622	206	2,720	148	20,222

Supplementary Table 2 Details of the populations analyzed in this study. AFR, African; EUR, European; MES, Middle-East; SAS, South Asian; CSA, Central and South Asia; EAS, East Asian; OCN, Oceanian; AMR, Americans; SCN, Southern Chinese. The HGDP samples were grouped by geographic region, and the sample sizes of subpopulations are shown in the brackets.

Population ID	Description	Group	Sample Size	Source
ACB	African Caribbeans in Barbados	AFR	96	KGP
ASW	Americans of African Ancestry in SW USA	AFR	61	KGP
ESN	Esan in Nigeria	AFR	99	KGP
LWK	Luhya in Webuye, Kenya	AFR	99	KGP
MSL	Mende in Sierra Leone	AFR	85	KGP
GWD	Gambian in Western Divisions in the Gambia	AFR	113	KGP
YRI	Yoruba in Ibadan, Nigeria	AFR	108	KGP
FIN	Finnish in Finland	EUR	99	KGP
GBR	British in England and Scotland	EUR	91	KGP
CEU	Utah Residents with Northern and Western European Ancestry	EUR	99	KGP
IBS	Iberian Population in Spain	EUR	107	KGP
TSI	Toscani in Italia	EUR	107	KGP
BEB	Bengali from Bangladesh	SAS	86	KGP
GIH	Gujarati Indian from Houston, Texas	SAS	103	KGP
ITU	Indian Telugu from the UK	SAS	102	KGP
PJL	Punjabi from Lahore, Pakistan	SAS	96	KGP
STU	Sri Lankan Tamil from the UK	SAS	102	KGP
CDX	Chinese Dai in Xishuangbanna, China	EAS	93	KGP
CHB	Han Chinese in Beijing, China	EAS	103	KGP
CHS	Southern Han Chinese	EAS	105	KGP
JPT	Japanese in Tokyo, Japan	EAS	104	KGP
KHV	Kinh in Ho Chi Minh City, Vietnam	EAS	99	KGP
CLM	Colombians from Medellin, Colombia	AMR	94	KGP
MXL	Mexican Ancestry from Los Angeles USA	AMR	64	KGP
PEL	Peruvians from Lima, Peru	AMR	85	KGP
PUR	Puerto Ricans from Puerto Rico	AMR	104	KGP
HGDP-AFR	BantuKenya (11), BantuSouthAfrica (8), Biaka (22), Mandenka (22), Mbuti (13), San (6), Yoruba (22)	AFR	104	HGDP
HGDP-EUR	Adygei (16), Basque (23), BergamoItalian (12), French (28), Orcadian (15), Oroqen (9), Russian (25), Sardinian (28), Tuscan (8)	EUR	155	HGDP
HGDP-MES	Bedouin (46), Druze (42), Mozabite (27), Palestinian (46)	MES	161	HGDP
HGDP-CSA	Balochi (24), Brahui (25), Burusho (24), Hazara (19), Kalash (22), Makrani (25), Pathan (24), Sindhi (24), Uygur (10)	CSA	197	HGDP
HGDP-EAS	Cambodian (9), Dai (61), Daur (9), Han (33), Hezhen (9), Japanese (27), Lahu (8), Miao (10), Mongolian (9), Naxi (8), NorthernHan (10), She (10), Tu (10), Tujia (9), Xibo (9), Yakut (25), Yi (10)	EAS	223	HGDP
HGDP-OCN	Bougainville (11), PapuanHighlands (9), PapuanSepik (8)	OCN	28	HGDP
HGDP-AMR	Colombian (7), Karitiana (12), Maya (21), Pima (13), Surui (8)	AMR	7	HGDP
GZ	Residents in Guizhou province, China	SCN	4082	Ref[1]
GX	Residents in Guangxi province, China	SCN	4834	Ref[1]
GD	Residents in Guangdong province, China	SCN	4622	Ref[1]
HN	Residents in Hainan province, China	SCN	2720	Ref[1]
YN	Residents in Yunnan province, China	SCN	3964	Ref[1]

Supplementary Table 3 Datasets and analytic tools used in this study.

Datasets/Tools	URL	Refs
1000 Genomes Project Phase III genome data	https://www.internationalgenome.org/	[2, 3]
Human Genome Diversity Project genome data	http://www.hagsc.org/hgdp/	[4]
JASPAR 2024	http://jaspar.genereg.net/	[5]
Eagle 2.4.1	https://data.broadinstitute.org/alkesgroup/Eagle	[6]
PLINK 1.9	http://zzz.bwh.harvard.edu/plink/	[7]
Haploview 4.2	https://www.broadinstitute.org/haploview/haploview	[8]
DnaSP 6.12.03	http://www.ub.edu/dnasp/	[9]
PopART 1.7	https://popart.otago.ac.nz	[10]
TMRCA calculator	https://github.com/Shuhua-Group/TMRCA/	[11]
rehh 2.0	https://cran.r-project.org/web/packages/rehh/index.html	[12]
Theta_D_H.Est	https://github.com/Shuhua-Group/Theta_D_H.Est/	[11]
RELATE 1.2.2	https://myersgroup.github.io/relate/	[13]
IGV 2.16.1	www.broadinstitute.org/igv/	[14]

Supplementary Table 4 Haplotypes of the β-globin locus with frequency > 0.2% in the SCN individuals.

Haplogroup	Haplotype	Frequency (%)	Sequence
HG1	H1	52.92	AGGGGAGCCTTCAAAGTACCCTGTATCTTCTCGACCCCCACGCC
	H2	10.96	AGGAGAGCCTTCAAAATACCCTGTATCTTCTCGACCCCCACGCC
	H3	8.04	AGGGGAGCCTTCAAAGTACCCTGTATCTTCGCGACCCCCACGCC
	H7	2.36	GGGAGAGCCTTCAAAATACCCTGTATCTTCTCGACCCCCACGCC
	H8	2.28	AGGAGAGCCTTCAAAGTACCCTGTATCTTCTCGACCCCCACGCC
	H9	0.48	AGGGGAGCCTTCAAAGTACCCTGTATCTTCTCGACCCCCACGCG
	H10	0.47	AGGGGAGCCTTCAAAGTACCCCGTATCTTCTCGACCCCCACGCC
	H13	0.22	AGGGGAGCCTTCAAAGTACCCTGCGCTACTTCGACGTTGGGATC
HG2	H11	0.38	GAAGATATTACGGTCGCTCCCCCTATCTTCTCGACCCCCACGCC
	H12	0.27	GAAGATATTACGGTCGCTCCCCCCGCTACTTCGACCCCCACGCC
	H5	6.10	GAAGATATTACGGTCGCTCCCCCCGCTACTTCGACGTTGGGATC
	H6	3.00	GAAGATATTACGGTCGCTCCCCCCGCTACTTCGACGCTGGGATC
HG3	H4	7.02	GAAGGTATTACGGTCGCTTAACGTGCTACCTTCCTGCCCGCATG
	H14	0.21	GAAGGTATTACGGTCGCTTAACGTGCTACCTCGACGCCCGCATG

Supplementary Table 5 Categorization of the haplotype heterogeneity of the 13 β-thalassemia mutations.

Category	β-thalassemia mutations	Description
1	CD71/72; CD27/28; IVS-II-5	Present in single haplotypes or haplogroups.
2	-50; Hb NewYork; -29; IVS-I-1	Shared by two haplogroups: HG1 and HG2 for IVS-I-1; HG1 and HG3 for the others.
3	CD41/42; CD17; IVS-II-654; CD43; -28; HbE	Shared by three haplogroups: the major haplogroups are HG2 for HbE, HG3 for -28, and HG1 for the others.

Supplementary Table 6 Number of haplotypes linked with the 13 commonly detected β-thalassemia mutations in the SCN samples.

Mutation	HG1	HG2	HG3
CD71/72	14	0	0
IVS-II-5	5	0	0
CD27/28	0	5	0
-28	7	1	79
CD41/42	410	5	3
CD17	174	3	1
IVS-II-654	85	2	3
CD43	3	2	1
HbE	21	35	3
Hb NewYork	28	0	1
-29	4	0	1
-50	40	0	14
IVS-I-1	1	9	0

Supplementary Table 7 Nucleotide diversity in haplotypes carrying the CD41/42 and -50 mutations across ethnic populations.

β-thalassemia mutation	Ethnic groups	Number of haplotypes	θ_π
CD41/42	GX-Han	58	14.61
	GX-Zhuang	71	14.46
	HN-Han	52	19.36
	HN-Li	44	13.28
	Other-SCN	194	16.38
-50	HN-Han	13	41.21
	HN-Li	6	4.80
	Other-SCN	34	17.45

Supplementary Table 8 Allele age estimates for the β-thalassemia mutations by RELATE. Ky, thousand years.

β-thalassemia mutation	Population	Age (Ky, Lower)	Age (Ky, Upper)
CD41/42	HN-Han	3.15	6.70
	HN-Li	4.05	7.08
	GX-Zhuang	4.48	7.42
	GX-Han	2.80	4.08
CD17	SCN	0.57	0.79
IVS-II-654	SCN	0.99	3.98
-28	SCN	0.26	3.66
HbE	SCN	0.57	2.69
-50	SCN	1.04	3.90
Hb NewYork	SCN	0.50	7.10

Supplementary Table 9 Predicted KLF1 motif scores for rs72872549.

JASPAR ID	Allele	Position (relative to rs72872549)	Sequence Context	Motif Match Score (Raw)	Motif Match Score (Relative)
MA0493.3	G	-1 to +6	GGGTGTAG	9.11	0.91
	G	-7 to 0	GGGTGAGG	7.22	0.87
	A	-1 to +6	GAGTGTAG	/	/
	A	-7 to +0	GGGTGAGA	/	/

Supplementary References

1. Shang X*, et al.* Rapid Targeted Next-Generation Sequencing Platform for Molecular Screening and Clinical Genotyping in Subjects with Hemoglobinopathies. *EBioMedicine* **23**, 150-159 (2017).

2. Fairley S, Lowy-Gallego E, Perry E, Flicek P. The International Genome Sample Resource (IGSR) collection of open human genomic variation resources. *Nucleic Acids Res* **48**, D941-D947 (2020).

3. The 1000 Genomes Project Consortium. A global reference for human genetic variation. *Nature* **526**, 68-74 (2015).

4. Bergstrom A*, et al.* Insights into human genetic variation and population history from 929 diverse genomes. *Science* **367**, (2020).

5. Rauluseviciute I*, et al.* JASPAR 2024: 20th anniversary of the open-access database of transcription factor binding profiles. *Nucleic Acids Res* **52**, D174-D182 (2024).

6. Loh P-R*, et al.* Reference-based phasing using the Haplotype Reference Consortium panel. *Nat Genet* **48**, 1443-1448 (2016).

7. Chang CC, Chow CC, Tellier LC, Vattikuti S, Purcell SM, Lee JJ. Second-generation PLINK: rising to the challenge of larger and richer datasets. *Gigascience* **4**, 7 (2015).

8. Barrett JC, Fry B, Maller J, Daly MJ. Haploview: analysis and visualization of LD and haplotype maps. *Bioinformatics* **21**, 263-265 (2005).

9. Rozas J*, et al.* DnaSP 6: DNA Sequence Polymorphism Analysis of Large Data Sets. *Molecular Biology and Evolution* **34**, 3299-3302 (2017).

10. Leigh JW, Bryant D. popart: full-feature software for haplotype network construction. *Methods in Ecology and Evolution* **6**, 1110-1116 (2015).

11. Pan Y*, et al.* Lineage-specific positive selection on ACE2 contributes to the genetic susceptibility of COVID-19. *Natl Sci Rev* **9**, nwac118 (2022).

12. Gautier M, Klassmann A, Vitalis R. rehh 2.0: a reimplementation of the R package rehh to detect positive selection from haplotype structure. *Molecular Ecology Resources* **17**, 78-90 (2017).

13. Speidel L, Forest M, Shi S, Myers SR. A method for genome-wide genealogy estimation for thousands of samples. *Nat Genet* **51**, 1321-1329 (2019).

14. Robinson JT, Thorvaldsdottir H, Turner D, Mesirov JP. igv.js: an embeddable JavaScript implementation of the Integrative Genomics Viewer (IGV). *Bioinformatics* **39**, (2023).
