# Supplementary material for: Multi-centric origins and gene flow shape the diversity of β-thalassemia mutations in Southern East Asia
Source: Nat Commun. 2025 Nov 20;16:10220. doi: 10.1038/s41467-025-65019-0 (PMC12635081; doi:10.1038/s41467-025-65019-0)
Supplement: Supplementary file 2 — Description of Additional Supplementary Files [file 41467_2025_65019_MOESM2_ESM.pdf]

Description of Additional Supplementary Files

Supplementary Data 1: Allele frequency and haplotype proportions of the 13 commonly detected β-thalassemia mutations in the SCN samples. For each mutation, haplotypes are sorted according to the proportions.
